# Association between social support and life space mobility in older patients after percutaneous coronary intervention: mediating roles of health literacy and intrinsic capacity

**DOI:** 10.3389/fpubh.2025.1726302

**Published:** 2026-01-05

**Authors:** Yue Dan, Qian Li, Ting Xu, Xuemei Tu, Li Yan

**Affiliations:** 1 School of Nursing, North Sichuan Medical College, Nanchong, Sichuan, China; 2 Department of Cardiovascular Medicine, Chongqing University Three Gorges Hospital, Chongqing, China; 3 Department of Teaching, Chongqing University Three Gorges Hospital, Chongqing, China

**Keywords:** health literacy, life space mobility, mediating effect, intrinsic capacity, percutaneous coronary intervention, social support

## Abstract

**Background:**

Enhancing life space mobility is instrumental in promoting healthy aging and improving the overall quality of life for older adults. However, further research is warranted to elucidate the determinants and the underlying mediating mechanisms that influence life space mobility in older patients following percutaneous coronary intervention (PCI).

**Objectives:**

Investigating the mediating roles of health literacy and intrinsic capacity in the relationship between social support and life space mobility among older patients following PCI.

**Methods:**

This cross-sectional observational study prospectively screened and consecutively enrolled 330 post-PCI patients (≥60 years) from the outpatient department of a tertiary hospital in Chongqing between January 2025 and September 2025. A general information questionnaire, the Perceived Social Support Scale, the Chronic Disease Health Literacy Scale, the Intrinsic Capacity Screening Form, and the Life Space Assessment Scale were used to collect data. The mediation model was tested using the SPSS PROCESS macro.

**Results:**

The life space mobility score among older patients following PCI was 58.12 ± 16.59. 51.8% of the patients were classified as having life space constriction. Correlation analyses revealed significant positive associations of social support, health literacy, and intrinsic capacity with life space mobility. The mediation analysis demonstrated a significant direct effect of social support on life space mobility. Furthermore, health literacy and intrinsic capacity were identified as significant partial mediators in this relationship. The specific proportions of the total effect mediated by health literacy and intrinsic capacity were 10.31 and 31.22%, respectively.

**Conclusion:**

Social support has an indirect impact on life space mobility through health literacy and intrinsic capacity, in addition to having a direct impact on post-PCI life space mobility in older patients. These findings underscore the importance of health literacy and intrinsic capacity as key modifiable factors. Developing comprehensive care strategies to enhance health literacy and intrinsic capacity is crucial for mitigating mobility decline and promoting successful recovery in older PCI patients.

## 1 Introduction

Coronary heart disease is a highly prevalent cardiovascular condition among older adults, characterized by high incidence and mortality rates, which significantly impair patients’ quality of life (1). Percutaneous coronary intervention (PCI) is a minimally invasive procedure for coronary revascularization. It involves dilating a stenotic or occluded coronary artery with a balloon and usually deploying a permanent stent to scaffold the vessel open, thereby improving blood supply to the ischemic myocardium (2). However, due to the persistent underlying pathology of atherosclerosis and ongoing cardiovascular risk factors, patients remain at risk for restenosis and the development of new lesions in other vascular segments following the procedure (3). Previous studies have demonstrated that cardiac rehabilitation after PCI helps prevent and treat coronary artery restenosis, reduces the disease burden, and improves long-term prognosis (4). With the rapid advancement of PCI technology, hospital stays have become shorter, necessitating continuous monitoring of cardiac rehabilitation outcomes by healthcare professionals after patient discharge. Life space mobility provides a simple and effective approach for monitoring cardiac rehabilitation following PCI (5).

Life space mobility refers to an individual’s capacity to move through various environments, ranging from their home to the broader community (6). It is measured by the spatial extent of movement, the frequency of trips, and the level of independence required to navigate these areas over a specified period. Life space mobility encompasses not only physical activity capabilities at the physiological level, but also comprehensively reflects the mental and psychological capacities of older adults, such as social participation and interpersonal interaction (7). Studies indicate that post-PCI patients often exhibit low levels of physical activity, with approximately 60.8% failing to meet physical activity recommendations outlined in cardiovascular guidelines (8). This physical inactivity directly contributes to life space constriction. Life space restriction, characterized by confinement to one’s immediate neighborhood, is operationally defined as a score below 60 on the Life Space Assessment (9). Hashimoto et al. (10) demonstrated that patients undergoing PCI often experience restricted life space, with an assessment score of 53.0 points serving as a significant threshold for predicting a higher risk of cardiovascular hospitalization within 2 years. Separately, a cross-sectional survey by Ishihara et al. (11) identified a moderate negative correlation between life space restriction and both cognitive decline and the prevalence of mild cognitive impairment (MCI) in patients with coronary heart disease, suggesting that life space restriction may be an important indicator for identifying MCI risk in this population. Furthermore, a broader body of evidence links restricted life space to various adverse health outcomes, including diminished mobility (12), an elevated risk of frailty (13), and a reduced quality of life (14). In the context of post-PCI recovery, Zhang et al. (15) identified pre-existing hypertension and low self-management capacity as significant risk factors for delayed recovery of life space mobility in older patients. However, the factors influencing life space mobility in older patients following PCI require further comprehensive investigation. This study aims to elucidate these factors to inform the development of targeted health management and personalized intervention strategies for addressing life space constriction in this population.

Social support refers to the multidimensional resources individuals obtain through their social networks (such as family, friends, neighbors, community organizations, etc.), specifically encompassing emotional care, material provision, informational support, and positive evaluation (16). Previous studies have indicated that life space mobility is positively impacted by social support, with patients who report higher levels of social support having better mobility outcomes (17). Lack of social support has several negative effects, especially for patients who have had PCI. These include a higher risk of depression (18), less adherence to cardiac rehabilitation (19), and a lower quality of life in terms of health (20). Currently, the mechanisms underlying the relationship between social support and life space mobility remain unclear, and the potential role of other mediating variables requires further investigation.

Health literacy refers to an individual’s ability to access, understand, and use health information to make sound health decisions (21). Higher health literacy not only aids in preventing the onset of cardiovascular disease but is also closely associated with improving adverse health outcomes. According to Eronen et al. (22), individuals with superior health literacy were more capable of participating in health promotion initiatives and making autonomous health decisions, which subsequently contributed to an expansion of their life space. Conversely, lower levels of health literacy can impede behavioral changes during cardiac rehabilitation (23), compromise self-management abilities (24), and elevate the risk of hospital readmission (25). Social support, serving as an external protective resource, can facilitate patients’ access to information and assistance, thereby fostering the development of their health literacy (26). While previous studies have established an association between health literacy and life space mobility, the mediating role of health literacy in the relationship between social support and life space mobility remains unexplored.

Intrinsic capacity is defined as the composite of all physical and mental capacities that an individual can draw upon at any point in time, encompassing five key domains: locomotion, cognition, vitality, sensory capacity (encompassing hearing and vision), and psychological health (27). Compared with the general older population, older patients with coronary heart disease demonstrate lower levels of intrinsic capacity and experience poorer clinical outcomes (28). Extensive research has established that impairments across multiple domains of intrinsic capacity—including cognitive decline (29), hearing loss (30), and depressive symptoms (31)—are associated with restricted life space mobility. The decline in intrinsic capacity is therefore considered a key driver of life space constriction. Furthermore, social support shows a positive correlation with intrinsic capacity (32), and targeted interventions providing social support have been shown to effectively mitigate or ameliorate the decline of intrinsic capacity in older adults (33). The literature identifies significant correlations among social support, health literacy, and intrinsic capacity with life space mobility. However, the pathways connecting these factors are not well characterized. To address this gap, this study employed a structural equation model to elucidate their interrelationships. The objective is to inform the development of targeted, multidimensional rehabilitation strategies designed to mitigate functional decline and improve long-term patient prognosis.

The health ecology model emphasizes that individual health arises from the reciprocal influence and interaction between personal and environmental factors, arranged from the innermost to the outermost levels as follows: innate personal characteristics, psychological and behavioral lifestyle, family and social networks, living and working conditions, and policy environment (34). As life space mobility is similarly shaped by these multi-level factors, the model enables a systematic analysis of its determinants. Social cognitive theory is a theoretical framework for guiding behavioral change, emphasizing the dynamic interaction among individual, environmental, and behavioral factors (35). This theory posits that individual behavioral change results from the combined influence of internal and external factors, being both self-determined and environmentally constrained. Specifically, individuals guide and control their behavior through innate traits and cognition, while changes in their environment also trigger behavioral modifications. Within the social cognitive theory framework, social support influences older adults’ life space mobility through cognitive factors (health literacy) and physiological factors (intrinsic capacity). Therefore, this study is grounded in the health ecology model and social cognitive theory, examines the dual mediating effects of social support and life space mobility on health literacy and intrinsic capacity in older patients following PCI. The research hypotheses are as follows:

*Hypothesis* 1: Social support can positively predict life space mobility.

*Hypothesis* 2: Health literacy mediates the relationship between social support and life space mobility.

*Hypothesis* 3: Intrinsic capability mediates the relationship between social support and life space mobility.

**Figure 1:**
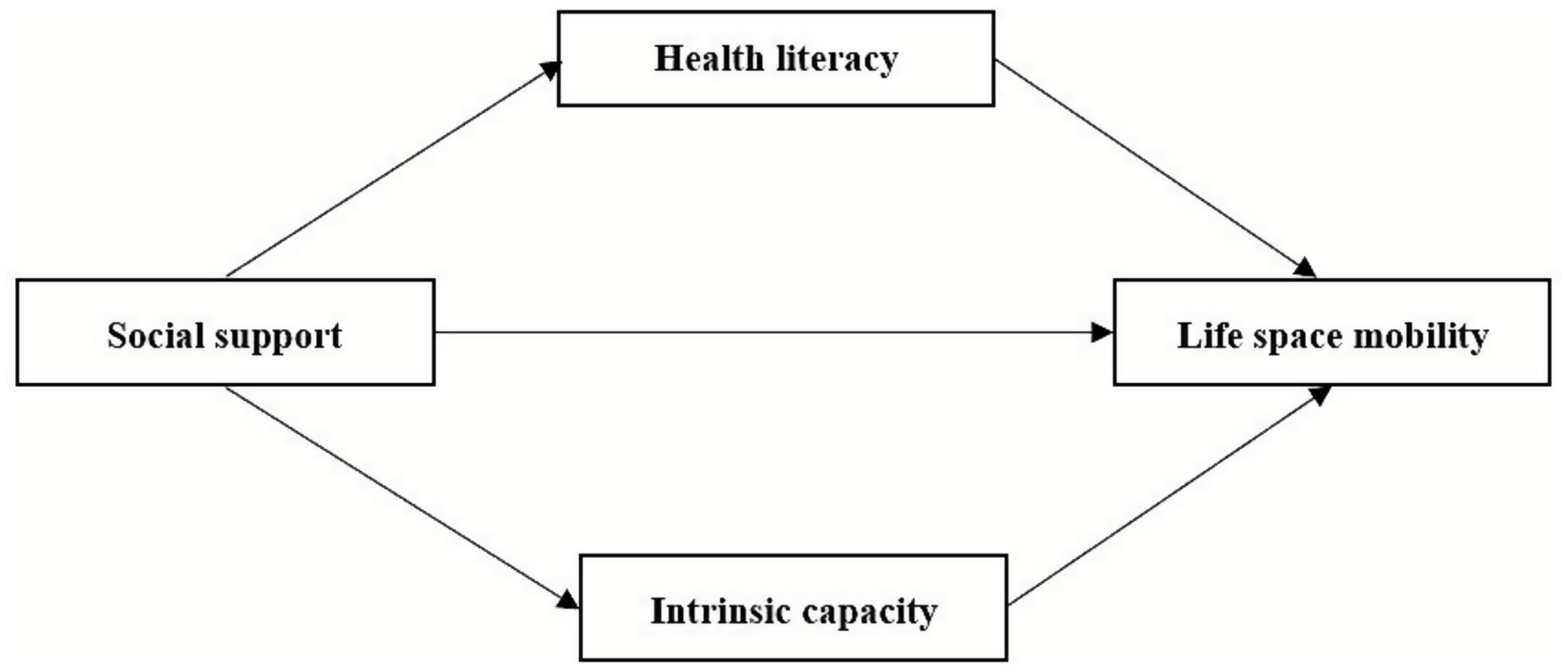
Hypothesis model influencing life space mobility in older patients after PCI.

The theoretical model hypothesized in this study is shown in Figure 1.

## 2 Method

### 2.1 Study design and participants

This study prospectively enrolled a consecutive sample of patients aged 60 years or older who were 1 to 2 months after PCI and presenting for follow-up at a tertiary hospital cardiology clinic in Chongqing between January and September 2025. The inclusion criteria were as follows: (a) age ≥60 years; (b) diagnosis of coronary atherosclerotic heart disease according to the New York Heart Association (NYHA) criteria (36) and having undergone PCI within the preceding 1–2 months; (c) ability to walk independently or with a walking aid; (d) conscious and able to provide cooperation for the study. Exclusion criteria included: (a) NYHA functional class IV; (b) critical illness or being in the acute phase of an illness; (c) comorbid severe cognitive impairment, psychiatric disorders, or significant visual/auditory impairments; (d) presence of severe complications or other conditions that substantially limit mobility.

The sample size was calculated using the formula for cross-sectional studies: $n=(\mu_{\alpha /2}^{2}\sigma^{2})\delta^{2}$ (37). A pre-survey of 30 cases yielded a standard deviation of 17.47 for patients’ life space mobility; based on this, δ was set to 2. A two-tailed test with α = 0.05 was used, which corresponds to a μα/2 value of 1.96. The initial sample size calculation yielded a result of 294 participants. To account for a potential 10% non-response rate, the required sample size was adjusted to 324. Ultimately, 330 participants were enrolled.

### 2.2 Measurement

#### 2.2.1 The general information questionnaire

A self-administered questionnaire was developed based on a comprehensive literature review. It collected data on the following sociodemographic and clinical characteristics: age, sex, literacy level, marital status, housing conditions, place of residence, average monthly income, body mass index, NYHA functional classification, history of hypertension, history of diabetes, history of hyperlipidemia, family history, and health insurance type.

#### 2.2.2 Perceived social support scale

This scale was developed by Zimet et al. (38) and adapted for the Chinese version by Jiang (39). It primarily assesses the level of support individuals perceive or experience from family, friends, and social relationships. Comprising 12 items, it is divided into three dimensions: family support, friend support, and other support. Responses are recorded on a 7-point Likert scale, resulting in a total score ranging from 12 to 84. Higher scores indicate a greater perceived level of social support. In the current study, the Cronbach’s *α* coefficient was 0.925.

#### 2.2.3 Chronic disease health literacy scale

This scale was developed by Jordan et al. (40) and adapted into a Chinese version by Sun et al. (41). This 24-item instrument comprises four dimensions: information acquisition ability (9 items), willingness to improve health (4 items), willingness to provide financial support (2 items), and communication and interaction ability (9 items). All items are rated on a 5-point Likert scale, yielding a total score that ranges from 24 to 120. A higher total score reflects a greater level of health literacy. In the current study, the Cronbach’s *α* coefficient was 0.945.

#### 2.2.4 Intrinsic capacity screening tool

A trained researcher assessed participants’ intrinsic capacity using a scale developed by the World Health Organization (WHO) in 2019, which is designed to rapidly identify areas of functional decline in older adults (42). The screening assesses five domains: cognition, mobility, nutrition, sensory capacity (visual and auditory), and psychological status, through a total of six items. The screening criteria for each domain were defined as follows: (1) Cognition: Impairment was indicated by failure to recall three words or an incorrect response to any orientation question; (2) Mobility: Limitation was defined as the inability to complete five chair rises within 14 s without using arm support; (3) Nutrition: Risk was identified by a weight loss of >3 kg in the past three months or a reported history of diminished appetite; (4) Sensory capacity: Loss was constituted by reported visual impairment (e.g., difficulty with distance vision or reading) or hearing impairment (e.g., failure in the whisper test); (5) Psychological status: Depression was defined as experiencing low mood, despondency, hopelessness, or a lack of interest/pleasure in activities over the past two weeks. Each unimpaired domain is scored 1 point, resulting in a total score ranging from 0 to 5, where a higher score denotes better intrinsic capacity. In the current study, the Cronbach’s *α* coefficient was 0.827.

#### 2.2.5 Life space assessment scale

This scale was developed by Baker et al. (43) and adapted into Chinese by Ji et al. (44) to assess life space mobility in older adults over the past 4 weeks. The scale comprises three dimensions: range of activities, frequency, and independence. The range of activities is subdivided into five zones: (1) areas beyond the bedroom but within the home; (2) areas outside the home but within the residential building; (3) areas within the residential compound but outside the building; (4) the local street immediately beyond the compound; (5) any other locations within the city. Activity frequency was rated using a four-point Likert scale: less than once per week (1 point); 1–3 times per week (2 points); 4–6 times per week (3 points); and daily (4 points). Activity independence was scored as: requiring assistance from another person (1 point); using an assistive device only (1.5 points); or requiring no assistance or device (2 points). The score for each area is calculated as ‘Activity Range × Frequency × Independence’. The sum of scores across all five areas constitutes the total score, ranging from 0 to 120. A higher score indicates greater life space mobility, while a total score <60 points suggests life space constriction. In the current study, the Cronbach’s *α* coefficient was 0.728.

### 2.3 Data collection

Before data collection, three nursing postgraduate students received standardized training, which covered the explanation of questionnaire content, the use of standardized guidance scripts, and protocols for providing completion assistance. Following approval from the hospital and relevant departments, the investigators distributed paper questionnaires face-to-face to eligible patients who met the inclusion and exclusion criteria. Standardized guidance scripts were utilized to explain the study purpose and questionnaire requirements, with uniform explanations provided for any items that patients found unclear. For respondents experiencing difficulty with reading or writing, investigators read each item aloud and recorded the responses on their behalf. All questionnaires were checked on-site for completeness before collection. All data were collected during a single outpatient follow-up visit scheduled 1–2 months after PCI. A total of 335 questionnaires were distributed, with 330 valid responses returned, resulting in a response rate of 98.5%. Five questionnaires were excluded due to logical inconsistencies in the provided data.

### 2.4 Statistical analysis

All statistical analyses were performed using IBM SPSS Statistics (version 26.0). The normality of quantitative data was assessed using Q-Q plots. Data conforming to a normal distribution are presented as mean ± standard deviation, while categorical data are summarized as frequencies and percentages. Relationships between variables were examined using Pearson’s correlation coefficients. Common method bias was evaluated using Harman’s single-factor test. Mediation analyses were conducted using Model 4 of the SPSS PROCESS macro (version 4.1), with a bootstrap sample of 5,000 to estimate effect sizes and their corresponding 95% confidence intervals. A two-sided *p*-value of less than 0.05 was considered statistically significant.

### 2.5 Ethical considerations

This study was conducted in accordance with the ethical principles of the Declaration of Helsinki and was approved by the Ethics Committee of Chongqing University Three Gorges Hospital (Approval No.: MR-50-25-012312). Written informed consent was obtained from all participants before their enrollment, after they had been fully informed about the study purpose, procedures, potential risks and benefits, and the measures for protecting their confidentiality. It was emphasized that participation was voluntary and that they could withdraw at any time without detriment to their medical care.

## 3 Results

### 3.1 Participant characteristics

The study population of 330 post-PCI patients was characterized by a predominance of men (58.8%), with half (50.9%) being in the 70–79 year age group, and a high prevalence of hypertension (57.3%). The mean life space mobility score for older PCI patients was 58.12 ± 16.59. Furthermore, 51.8% of the patients were classified as having restricted life space mobility. Univariate analysis identified several factors significantly associated with reduced life space mobility, including advanced age, female sex, lower education level, being unmarried, living alone, lower monthly household income, higher NYHA class, hypertension, and diabetes (*p* < 0.05). No such associations were found for residence, BMI, hyperlipidemia, family history, or insurance type (*p* > 0.05), as detailed in Table 1.

**Table 1:** Distribution of demographic and illness characteristics and the associations with life space mobility (*n* = 330).

Variable	N (%)	Score (mean ± SD)	*T*/*F*	*p* value
Age (years)			40.179	<0.001
60 ~ 69	101(30.6)	67.87 ± 15.43		
70 ~ 79	168(50.9)	56.35 ± 14.51		
≥80	61(18.5)	46.86 ± 15.11		
Sex			3.099	0.002
Male	194(58.8)	60.46 ± 15.67		
Female	136(41.2)	54.78 ± 17.32		
Literacy level			11.175	<0.001
Primary and below	190(57.6)	54.57 ± 15.47		
Junior high school	86(26.1)	62.17 ± 16.22		
High school and above	54(16.3)	64.18 ± 17.99		
Marital status			4.311	<0.001
Married	260(78.8)	60.11 ± 16.45		
Unmarried	70(21.2)	50.73 ± 15.00		
Housing conditions			−2.758	0.006
Living alone	50(15.2)	52.22 ± 14.14		
Not living alone	280(84.8)	59.17 ± 16.79		
Place of residence			−1.028	0.305
Rural	142(43.0)	57.06 ± 15.06		
City	188(57.0)	58.92 ± 17.65		
Average monthly income (yuan)			4.932^a^	0.008
<1,000	79(23.9)	54.95 ± 14.58		
1,000 ~ 3,000	141(42.7)	56.88 ± 15.40		
>3,000	110(33.4)	61.98 ± 18.69		
BMI(kg/m^2^)			1.482	0.219
<18.5	15(4.5)	54.13 ± 18.78		
18.5 ~ 23.9	157(47.6)	56.80 ± 15.31		
24 ~ 27.9	117(35.4)	60.56 ± 17.75		
≥28	41(12.5)	57.68 ± 16.77		
Heart function classification			52.431	<0.001
Class I	162(49.1)	65.15 ± 13.90		
Class II	124(37.6)	54.96 ± 15.02		
Class III	44(13.3)	41.11 ± 15.01		
History of hypertension			−2.658	0.008
Yes	189(57.3)	56.04 ± 17.20		
No	141(42.7)	60.90 ± 15.35		
History of diabetes			−3.280	0.001
Yes	78(23.6)	52.56 ± 17.46		
No	252(76.4)	59.84 ± 15.95		
History of hyperlipidemia			1.291	0.198
Yes	37(11.2)	61.43 ± 14.31		
No	293(88.8)	57.70 ± 16.83		
Family history			0.838	0.403
Yes	46(13.9)	60.02 ± 17.09		
No	284(86.1)	57.81 ± 16.51		
Health insurance type			1.757	0.080
New rural cooperative medical scheme	78(23.6)	60.99 ± 15.23		
Employee medical insurance / Resident medical insurance	252(76.4)	57.23 ± 16.91		

BMI, body mass index; NYHA, New York Heart Association; CHD, coronary heart disease; SD, standard deviation.

a Welch’s test for F-value when variance is not equal.

### 3.2 Correlation analysis among variables

Pearson correlation analysis revealed that life space mobility was positively correlated with social support (r = 0.518, *P*<0.01), health literacy (r = 0.471, *P*<0.01), and intrinsic capacity (r = 0.637, *P*<0.01). Social support was positively correlated with health literacy (r = 0.471, *p* < 0.01) and intrinsic capability (r = 0.521, *p* < 0.01). Health literacy was positively correlated with intrinsic capability (r = 0.384, *p* < 0.01). The correlation coefficients among variables are presented in Table 2.

**Table 2:** Correlation analysis of social support, health literacy, intrinsic capacity, and life space mobility (r-values).

Variable	Social support	Health literacy	Intrinsic capacity	Life space mobility
Social support	1			
Health literacy	0.471^**^	1		
Intrinsic capacity	0.521^**^	0.384^**^	1	
Life space mobility	0.518^**^	0.471^**^	0.637^**^	1

**P < 0.01.

### 3.3 Constructing structural equation models

Potential common method bias was assessed using Harman’s single-factor test. The results showed that 12 factors had eigenvalues greater than 1. The first factor accounted for 29.26% of the variance, which is below the critical threshold of 40%, indicating that there was no significant standard method bias in this study.

**Figure 2:**
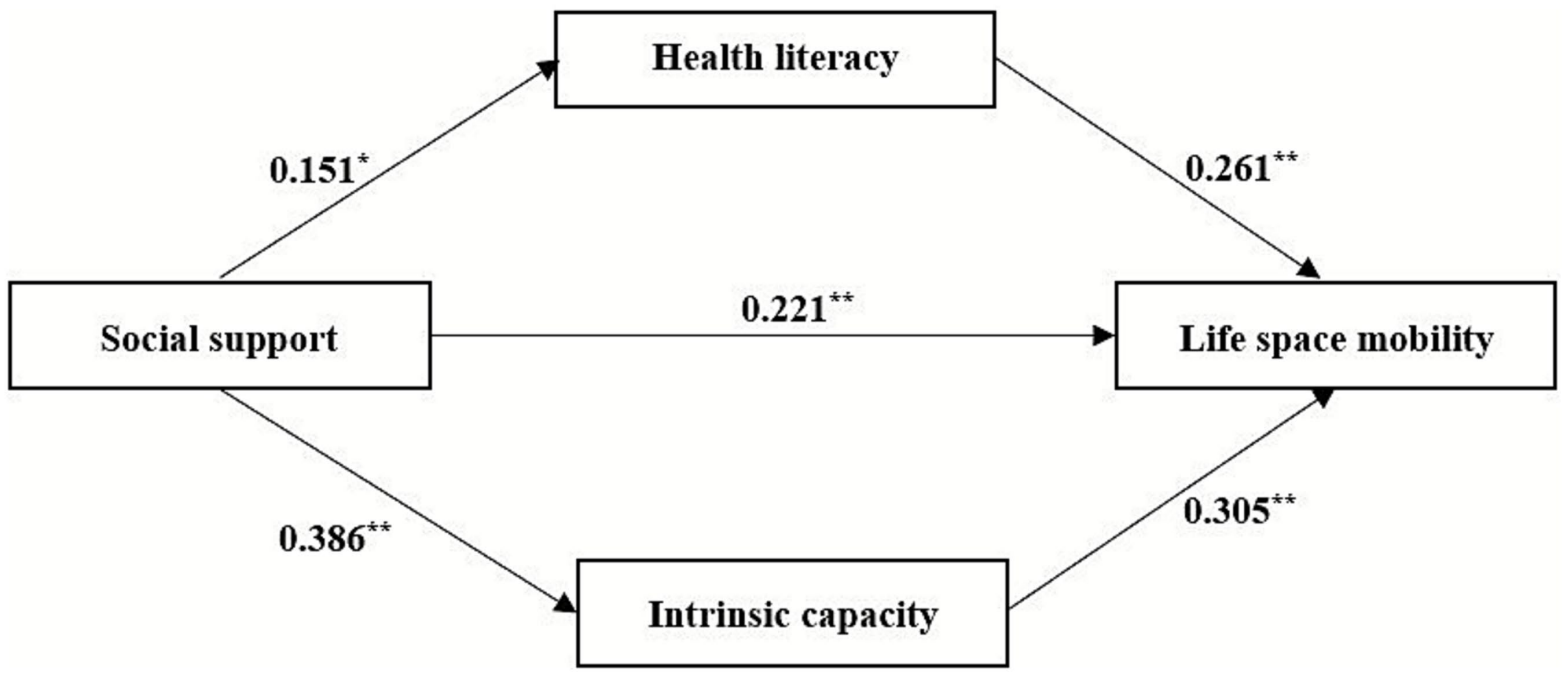
The parallel mediating role of health literacy and intrinsic capacity on the chain between social support and life space mobility. ^*^p < 0.05; ^**^p < 0.01.

To test the hypothesized model, data were analyzed using Model 4 of the SPSS PROCESS macro, controlling for all covariates that were significant in the univariate analysis. The model defined social support as the independent variable, life space mobility as the dependent variable, and both health literacy and intrinsic capacity as simultaneous mediators. The mediational analysis yielded a significant direct effect of social support on life space mobility (*β* = 0.221, *p* < 0.001). Moreover, significant mediation effects were observed. Specifically, social support had a significant positive effect on health literacy (*β* = 0.151, *p* = 0.012), and health literacy subsequently had a significant positive effect on life space mobility (*β* = 0.261, *p* < 0.001). Likewise, social support significantly and positively influenced intrinsic capacity (*β* = 0.386, p < 0.001), and intrinsic capacity thereby significantly and positively influenced life space mobility (*β* = 0.305, *p* < 0.001). The comprehensive results are depicted in Figure 2 and summarized in Table 3.

**Table 3:** Path coefficients between variables.

Dependent variable	Independent variable	B	SE	t	95%CI
Life space mobility	Social support	0.378	0.105	6.522^**^	0.480 ~ 0.895
Health literacy	Social support	0.151	0.094	2.529^*^	0.053 ~ 0.421
Intrinsic capacity	Social support	0.386	0.011	6.508^**^	0.048 ~ 0.090
Life space mobility	Social support	0.221	0.102	3.948^**^	0.202 ~ 0.602
Life space mobility	Health literacy	0.261	0.057	5.291^**^	0.191 ~ 0.416
Life space mobility	Intrinsic capacity	0.305	0.506	6.134^**^	2.110 ~ 4.103

B, standardized coefficients; SE, standard error; CI, confidence interval. ^*^P < 0.05; ^**^P < 0.01.

The mediation effects were tested using the bootstrap method with 5,000 bootstrap samples and a 95% confidence interval. Bootstrap test results (Table 4) demonstrate that social support has a significant direct effect on life space mobility, with an effect size of 0.221, which accounts for 58.47% of the total effect. The indirect effects are as follows: (1) For the path “Social Support → Health Literacy → Life Space Mobility,” the 95% confidence interval does not include zero, indicating a significant mediating effect of health literacy. The effect size is 0.039, accounting for 10.31% of the total effect. (2) For the path “Social Support → Intrinsic Capacity → Life Space Mobility,” the 95% confidence interval does not include zero, suggesting a significant mediating effect of intrinsic capacity. The effect size is 0.118, accounting for 31.22% of the total effect. In summary, these results demonstrate that social support influences life space mobility through multiple pathways. Both health literacy and intrinsic capacity play important mediating roles, with intrinsic capacity emerging as the relatively more prominent mediator in this process.

**Table 4:** Percentage of path coefficients.

Effect	*β*	SE	95%CI	Effect proportion (%)
Total effect	0.378	0.105	0.480 ~ 0.895	100.00
Direct effect	0.221	0.102	0.202 ~ 0.602	58.47
Total indirect effect	0.157	0.030	0.101 ~ 0.219	41.53
Indirect effect 1	0.039	0.020	0.003 ~ 0.082	10.31
Indirect effect 2	0.118	0.024	0.074 ~ 0.168	31.22

B, standardized coefficients; SE, standard error; CI, confidence interval. Indirect effect 1: Social support → Health literacy → Life space mobility. Indirect effect 2: Social support → Intrinsic capacity → Life space mobility.

## 4 Discussion

This study investigated the pathway relationship between social support and life space mobility in older patients following PCI. By analyzing the interrelationships among basic demographic characteristics, social support, health literacy, intrinsic capacity, and life space mobility, this study identified key factors influencing life space mobility and elucidated the underlying mechanisms.

### 4.1 Current status and influencing factors of life space mobility

The findings of this study indicate that older patients who underwent PCI had a life space mobility score of 58.12 ± 16.59, corresponding to a moderate-to-low overall level. The rate of life space constriction was 51.8%. The prevalence of restricted life space observed in this study was higher than that reported by Moored et al. (45) in older men with osteoporotic fractures (18.0%). This discrepancy can be attributed to differences in the clinical and demographic profiles of the study populations. Post-PCI older patients frequently experience physical symptoms such as fatigue, pain, or swelling, which can diminish their motivation for activity and subsequently lead to life space restriction. Additionally, while the cohort of Moored et al. (45) was exclusively male, evidence suggests that males generally maintain higher levels of life space mobility than females (46). This sex disparity may be influenced by socio-cultural norms, wherein males often have stronger social incentives for outdoor engagement, whereas females may assume more domestic and caregiving roles, resulting in activity patterns that are more home-centric. Consequently, healthcare professionals should pay increased attention to female patients and encourage their participation in group-based outdoor activities to enhance social engagement and effectively improve their life space mobility.

Univariate analysis revealed that age, sex, educational attainment, marital status, housing conditions, monthly household income per capita, history of hypertension, history of diabetes mellitus, and heart function classification significantly influenced life space mobility in older patients following PCI (*p* < 0.05). Potential causes: (1) As people age, their physiological reserves, including their heart and lungs, muscles, and balance, naturally deteriorate (47). This results in a reduction in physical capabilities that limits their mobility and hastens the onset of life space constriction. (2) Patients with lower educational levels may have a limited capacity to comprehend disease-related knowledge and insufficient awareness of their own health status, thereby increasing the risk of life space constriction. (3) Patients lacking a spouse or living alone are susceptible to significant loneliness due to the absence of psychological support from an intimate partner, which reduces their willingness for outdoor activities and level of social engagement. In contrast, married patients within stable family structures (e.g., living with a spouse or children) receive more timely detection of and attention to changes in their emotional and health status. This close-knit family network serves as a protective factor, effectively assisting them in maintaining and expanding their life space by providing continuous emotional and instrumental support. (4) Higher monthly household income per capita facilitates private car ownership, enabling convenient travel to distant locations and directly expanding the geographical scope of activities. Conversely, financial constraints may cause patients from lower-income households to delay or forgo essential medical follow-ups and cardiac rehabilitation, thereby impeding functional recovery and exacerbating life space constriction. (5) In this study, a high proportion of older coronary heart disease patients had concomitant hypertension (57.3%). The coexistence of coronary heart disease and hypertension synergistically increases the cardiac workload. This elevated workload raises myocardial oxygen consumption and promotes left ventricular remodeling and hypertrophy, thereby accelerating the onset and progression of heart failure. The symptoms of heart failure, such as reduced exercise tolerance and fatigue, directly limit patients’ daily activity ranges (48). Additionally, patients with comorbid diabetes often develop peripheral neurovascular complications, which manifest as weakened knee extensor muscle strength, pain upon activity, or abnormal plantar sensation. These factors collectively lead to lower limb dysfunction, further restricting the patients’ life space (49). (6) Cardiac function classification reflects the heart’s pumping capacity and disease severity. Patients with poorer cardiac function often experience pronounced dyspnea, persistent fatigue, and reduced exercise tolerance, which severely limit their daily activities and mobility (50). Furthermore, during cardiac rehabilitation, those with higher cardiac function classifications are susceptible to exercise phobia, characterized by excessive vigilance toward activity-induced symptoms and avoidance of physical exertion (51). This behavior can lead to physical deconditioning and potentially worsen cardiac impairment, thereby intensifying life space constriction. Therefore, healthcare professionals should assess patients’ cardiac function and exercise tolerance during cardiac rehabilitation. Based on this assessment, individualized and progressive exercise programs should be developed and implemented to improve functional capacity. Additionally, it is essential to enhance health education and psychological support by instructing patients on cardiac rehabilitation and encouraging active participation in rehabilitation exercises, thereby promoting the expansion of life space mobility.

### 4.2 Social support can positively predict life space mobility

A significant positive correlation between social support and life space mobility was identified in this study among older post-PCI patients, which aligns with the findings reported by Hanlon et al. (52). Social support is defined as an individual’s subjective perception of the availability of assistance from family, friends, and other social connections (16). In chronic disease management, it serves as a key facilitator for establishing and maintaining healthy behaviors. Older patients with higher levels of social support receive more emotional and material assistance, which enhances their motivation to engage in rehabilitation training. This improved motivation subsequently improves physical function and ultimately expands life space (52). Kuspinar et al. (53) further established social support as a significant predictor of life space mobility in older adults, substantially reducing the risk of restricted life space. A qualitative study also revealed that family support is a key environmental facilitator for functional activities (54). When families provide more comprehensive assistance with daily living, healthcare support, and necessary environmental modifications, patients’ life space mobility improves accordingly. A robust family support system serves as a cornerstone for motivating patients’ active engagement in cardiac rehabilitation. A systematic review by Popescu et al. (55) confirmed that family-provided support—encompassing continuous behavioral monitoring (e.g., ensuring medication adherence, regular exercise, and a balanced diet) and emotional encouragement—significantly improves patient compliance. This enhanced adherence, in turn, yields substantial benefits across physiological function, psychological well-being, and overall quality of life. Therefore, healthcare professionals should emphasize the role of social support in rehabilitation and actively encourage family involvement in rehabilitation plans. By offering greater emotional care and daily companionship, along with encouraging and assisting patients in regular, individualized rehabilitation training, a positive rehabilitation cycle can be established.

### 4.3 The mediating role of health literacy

This study identified health literacy as a mediator in the relationship between social support and life space mobility in older patients after PCI. According to the social support buffering theory (56), when individuals face stressful events such as coronary heart disease and PCI surgery, robust social support helps them adjust their cognition and behavior to promote health. Specifically, patients with greater social support are better able to utilize social resources to access and comprehend healthcare information, thereby directly enhancing their health literacy (26). Higher health literacy subsequently motivates patients to proactively communicate with healthcare providers and adhere to healthy lifestyles. These positive health behaviors ultimately lead to improved life space mobility (57). Conversely, inadequate social support exacerbates feelings of helplessness and depression in older patients (18). This not only reduces their intrinsic motivation to acquire health knowledge and utilize information but also interacts with their inherently low health literacy. Together, these factors lead to the adoption of more passive coping strategies during rehabilitation (58) and self-restriction of daily activities due to excessive worry and fear about their condition (59), ultimately resulting in significantly constrained life space mobility. A prospective cohort study demonstrated that participation in cardiac rehabilitation was associated with a significant improvement in patients’ health literacy. This enhancement is achieved through structured programs that bolster knowledge of disease management and foster the adoption and maintenance of healthy behaviors (60). Healthcare professionals should therefore guide patients in establishing and maintaining scientifically grounded health behaviors through structured cardiac rehabilitation education, thereby facilitating life space expansion. Additionally, peer support models can be implemented by inviting patients with successful recovery outcomes to share their experiences. This approach provides both practical knowledge and emotional support, helping patients with limited health literacy to build rehabilitation confidence and improve their overall recovery quality.

### 4.4 The mediating role of intrinsic capacity

This study identified intrinsic capacity as a mediator in the relationship between social support and life space mobility in older patients following PCI. According to the healthy ageing model (61), an individual’s functional performance depends on the interaction between their intrinsic capacity and environmental characteristics. As a key element of the external environment, social support can effectively enhance life space mobility when well-aligned with an individual’s intrinsic capacity. Emotional support, material assistance, and daily care provided by children and other relatives can improve older patients’ social participation and daily functioning, thereby helping to maintain their intrinsic capacity (62). A higher level of intrinsic capacity indicates that older adults possess greater resources and capability reserves, enabling them to cope more effectively with fatigue and mitigate the risk of declined physical activity (63). Conversely, inadequate social support is associated with limited social networks and a reluctance to seek help, factors that may accelerate disease progression and contribute to a decline in intrinsic capacity (64). The decline in intrinsic capacity—manifested as reductions in muscular strength, sensory function, and cognitive ability—further restricts patient mobility, thereby directly and negatively impacting life space mobility (65). Furthermore, Pua et al. (66) demonstrated that a decline in intrinsic capacity not only directly contributes to life space mobility restrictions but also indicates an increased risk of frailty and sarcopenia. In older patients following PCI, intrinsic capacity has a greater direct impact on improving life space mobility than health literacy, due to their distinct mechanisms of action. Health literacy primarily influences outcomes by promoting self-care and adherence to rehabilitation programs—factors that are crucial but operate indirectly. Conversely, intrinsic capacity encompasses the comprehensive integration of an individual’s physical and mental capabilities, directly determining the upper and lower limits of functional activity. Since the decline in intrinsic capacity is a dynamic and reversible early-stage process, targeting it as a core focus allows for the early identification of multidimensional decline before functional dependency occurs, thus enabling precise intervention. Consequently, healthcare providers should enhance the screening for intrinsic capacity in older patients and expand care priorities from solely treating diseases to comprehensively optimizing intrinsic capacity. This approach helps delay the onset of mobility limitations and promotes healthy aging. Through multidisciplinary interventions—including health education, cognitive training, multicomponent physical exercise, and dietary guidance (67)—patients’ intrinsic capacity in domains such as mobility, cognition, and vitality can be enhanced. This, in turn, strengthens their health beliefs and fosters the maintenance of healthy behaviors.

### 4.5 Limitations

However, this study has several limitations that warrant consideration: (1) This study recruited patients from a single center only, which may limit the representativeness of the sample and the generalizability of the findings. (2) The use of a cross-sectional design inherently limits the inference of causality, as it can only identify associations between variables rather than establish causal relationships. (3) The Life Space Assessment Scale, which assesses self-reported activity areas over the past month, may be susceptible to recall bias. (4) While life space mobility is influenced by multiple factors, this study focused exclusively on the roles of social support, health literacy, and intrinsic capacity. Consequently, future research should investigate other potential determinants, such as psychological factors, to provide a more comprehensive understanding.

## 5 Conclusion

The overall life space mobility among older patients following PCI was observed to be at a moderate-to-low level, suggesting a significant potential for enhancement. Social support was significantly associated with life space mobility, both directly and indirectly through the mediating effects of health literacy and intrinsic capacity. These three factors represent key modifiable targets for clinical intervention. Future research should therefore focus on designing and evaluating targeted, multimodal interventions (such as peer support, health education, cognitive training, and dietary guidance) that address this entire pathway to effectively improve functional mobility outcomes in this patient population.

## Data Availability

The raw data supporting the conclusions of this article will be made available by the authors, without undue reservation.
